# Effect of the uncoupling protein-2 (UCP-2) and nuclear receptor subfamily 3 group C member 1 (NR3C1) genes on treatment efficacy and survival in patients with multiple myeloma: a single-center study

**DOI:** 10.1186/s13104-021-05758-7

**Published:** 2021-09-04

**Authors:** Ilknur Demir, Sacide Pehlivan, Vahap Okan, Handan Haydaroglu Sahin, Salih Sertaç Durusoy, Istemi Serin, Yasemin Oyaci, Mustafa Pehlivan

**Affiliations:** 1 Department of Hematology, Faculty of Medicine, Gaziantep University, Gaziantep, Turkey; 2 Department of Internal Medicine, Faculty of Medicine, Gaziantep University, Gaziantep, Turkey; 3 Department of Medical Biology, Istanbul Faculty of Medicine, Istanbul University, Istanbul, Turkey; 4 Department of Hematology, University of Health Sciences, Istanbul Training and Research Hospital, Org. Nafiz Gurman Cad., Fatih, 34098 Istanbul, Turkey

**Keywords:** Multiple myeloma, UCP-2, NR3C1, Prognosis, Autologous stem cell transplantation, Overall survival

## Abstract

**Objective:**

Studies on the genetic background of patients with multiple myeloma (MM) have been increasing; two important factors considered in such works are uncoupling protein-2 (UCP-2) and nuclear receptor subfamily 3 group C member 1 (NR3C1). We aim to reveal the association of MM with NR3C1 and UCP-2 gene polymorphisms. In this prospective study, 200 patients diagnosed between January 2009 and 2018 and 200 healthy individuals were included. For patients who had undergone autologous stem cell transplantation and control subjects, we statistically compared the CC, GC, and GG genotypes and the C and G alleles of the NR3C1 gene, as well as the AA, AG, and GG genotypes and the A and G alleles of the UCP-2 gene.

**Results:**

While the AA genotype was significantly more common in the MM group (p = 0.001), the GG genotype was significantly more common in the control group (p = 0.016). Overall survival was found to be significantly shorter in patients with the UCP-2 GG genotype (p = 0.034). It was also found that having the GG genotype of the UCP-2 gene was a 2.48-fold risk factor for mortality. The fact that overall survival is significantly shorter in MM patients with the UCP-2 GG genotype and its definition as a risk factor for mortality have been put forward for the first time in the literature.

**Supplementary Information:**

The online version contains supplementary material available at 10.1186/s13104-021-05758-7.

## Introduction

Multiple myeloma (MM) is a malignant disease of plasma cells that causes overproduction of monoclonal light and heavy chains [1]. High-dose chemotherapy followed by autologous stem cell transplantation (ASCT) is the preferred standard therapy for eligible patients with a diagnosis of MM. The International Staging System (ISS) was created with beta-2 microglobulin (β2-MG) and serum albumin values. In addition to the ISS, the Revised ISS (R-ISS) was created by using additional factors such as serum lactate dehydrogenase (LDH) and deletion 17p, t (4; 14), t (14; 16) detected by interphase fluorescent in situ hybridization (FISH) [2–4].

Treatment preferences and the relationships between treatment resistance and genetic infrastructures are frequent subjects of new studies. Among these, two important genetic factors are uncoupling protein-2 (UCP-2) and nuclear receptor subfamily 3 group C member 1 (NR3C1). UCPs are members of the mitochondrial anion transporter superfamily. There are five known types of UCP, referred to as UCP-1 through UCP-5 [5]. UCP-2 is widely expressed in cancer cells and can alleviate oxidative stress by suppressing mitochondrial production of reactive oxygen species (ROS). Loss of UCP-2 function can increase ROS production, while its overexpression may support cytoprotection by reducing oxidative stress [6]. Additionally, UCP-2 plays a role in carcinogenesis and chemoresistance [7–10]. Data on hematological malignancies and UCP-2 are limited. The effect of ROS production, which is the mechanism of many chemotherapeutic agents, and especially of melphalan and cyclophosphamide, used in the treatment of MM as alkylating agents, may be associated with treatment resistance. Therefore, the relationship between UCP-2 and MM seems to be worth investigating.

NR3C1 is the gene encoding the glucocorticoid receptor (GR). Glucocorticoids (GCs) are steroid hormones that exert pro- or anti-apoptotic effects through changes in GR and NR3C1 gene expression [11, 12]. Although it is known as a potent inducer of apoptosis in lymphoid cells, only a fraction of the signaling pathways that regulate the sensitivity or resistance of cancer cells to GCs have been identified [13–16].

The main aim of this study is to reveal the relationship between UCP-2 and NR3C1 polymorphisms and the prognosis and survival of MM patients who have undergone autologous stem cell transplantation (ASCT).

## Main text

### Patients and methods

In this study, 200 patients diagnosed with MM in the Gaziantep University Hematology Clinic between January 2009 and January 2018 and another 200 healthy individuals were included. In addition to demographic data, such as age and gender, the patients’ initial Durie-Salmon stages, International Staging System (ISS) scores, Eastern Cooperative Oncology Group (ECOG) scores, laboratory data (hemoglobin, leukocytes, platelets, C-reactive protein (CRP), lactate dehydrogenase (LDH), β2-MG, albumin), first-line treatments, overall survival (OS) and progression-free survival (PFS) data, mortality rates, and mean follow-up duration were recorded.

All patients were found eligible for ASCT at the initial evaluation. ASCT was performed for 77.5% of patients after 4 courses of VCD (bortezomib, cyclophosphamide, and dexamethasone) with at least a partial remission (PR) and then LD (lenalidomide and dexamethasone) was used as maintenance therapy for the following 24 months.

The CC, GC, and GG genotypes and the C and G alleles of the NR3C1 gene were statistically compared before treatment between patients having undergone ASCT and healthy controls, as were the AA, AG, and GG genotypes and the A and G alleles of the UCP-2 gene. Additionally, the statistically significant effects of these genotypes on PFS and OS in patients after ASCT were examined. The study was begun after obtaining the approval of the Gaziantep University Ethics Committee (07-2007/40).

### Isolation of genetic material

Genomic DNA was isolated from leukocytes with a GenMark isolation kit. The rs41423247 variants of NR3C1 gene and rs659366 variants belonging to the UCP-2 gene were respectively studied using the PCR–RFLP method [17, 18] from DNA samples obtained from the peripheral blood samples of individuals.

### Statistical analysis

The SPSS 21 package program was used for the statistical analysis of all data. The statistical significance of the differences between the patient and control groups was estimated by logistic regression analysis. Exp odds ratios (ORs) were calculated with a logistic regression model controlled for gender and age and were reported at 95% confidence intervals. Differences in NR3C1 and UCP-2 allele and genotype frequencies between the control and patient groups were compared using the χ^2^ test and, when needed, Fisher’s exact test was used. The Hardy–Weinberg equation was used to calculate estimated genotype frequencies and observed genotype frequencies. Survival probabilities were estimated by the Kaplan–Meier method and differences were compared using the log rank test. Cox stepwise regression analysis was employed to confirm the significance of risk factors. In multivariate analysis, we used eliminated variables stepwise (backward) with significance of less than 10%. Values of p < 0.05 were considered to indicate statistical significance.

### Results

The median age of the patient group was 56 years (range: 28–81). The 5-year OS of the patients was 71% with a median of 66 months, while the 5-year PFS was 44% with a median of 43.8 months. Mortality was 26% with a total of 46 deceased patients **(**Table 1).

**Table 1:** Clinical features and treatment regimens of MM patients

1	2	3. MM	4. Control
Age, median (range)		56 (28–81)	53 (22–68)
Gender, n (%)	Female/Male	90/110 (45/55)	99/101 (50/50)
Ig subtype, n (%)	IGG k	61 (30.5)	
	IGG l	30 (15)	
	IGA k	48 (24)	
	IGA l	27 (13.5)	
	Light chain (k/l)	34 (17/17)	
Stage (Durie-Salmon), n (%)	II/III	53/117 (31/69)	
	A/B	129/41 (76/24)	
ISS, n (%)	I	51 (30.2)	
	II/III	45/73 (26.6/43.2)	
ECOG, n (%)	> 1	28/169 (16.6)	
Hemoglobin	g/dL (range)	10.4 (5.9–15.8)	
Leukocytes	/mm^3^	6785 (2760–18,500)	
Thrombocytes	10^3^/mm^3^	172 (28–788)	
CRP	mg/dL	8.0 (2.1–342)	
LDH	IU/L	200 (93–1037)	
β2-MG	mg/L	5.0 (1.5–47)	
Albumin	g/L	3.5 (1.6–5.1)	
Treatment, n (%)	VCD, ASCT, LD		
	VCD ± LD		
OS, median, months (%)		66 (71)	
PFS, median, months (%)		43.8 (44)	
Mortality, n (%)			
Follow-up period, median, months (range)		30 (4.8–155)	

*MM* Multiple myeloma, *CD* bortezomib, cyclophosphamide, dexamethasone, *LD* lenalidomide, dexamethasone, *ASCT* autologous stem cell transplantation, *ECOG* Eastern Cooperative Oncology Group performance status, *CRP* C-reactive protein, *LDH* lactate dehydrogenase, *IPI* International Prognostic Index, *PFS* progression-free survival, *OS* overall survival

The AA genotype of UCP-2 was significantly more common in the MM group (p = 0.001), while the GG genotype was significantly more common in the healthy control group (p = 0.013). Similarly, the G allele was found to be significantly more common in the healthy control group (p = 0.001) (Table 2).

**Table 2:** Comparison of UCP-2 gene variants between all MM patients and the healthy control group

	Genotype	MM	Healthy Controls	OR Exp(B)	95% CI	p^&^
		n (%)	n (%)			
UCP-2	AA	92 (46)	32 (16)	0.813	0.116–0.411	0.001*
	AG	86 (43)	125 (62.5)	0.944	0.413–1.322	0.093
	GG	22 (11)	43 (21.5)	0.855	0.164–0.889	0.036*
Allele						
	A	270 (67.5)	189 (47.25)			
	G	130 (32.5)	211 (52.75)	0.667	0.355–0.699	0.001*

*MM* Multiple myeloma, *ASCT* autologous stem cell transplantation, *statistical significance

In the statistical analysis performed for the NR3C1 genotypes and alleles, no significant difference was found between the MM patients and the healthy control group (p > 0.05 for all) (Table 3).

**Table 3:** Comparison of NR3C1 gene variants between all MM patients and the healthy control group

	Genotype	MM	Healthy Controls	OR Exp(B)*	95% CI	p^&^
		n (%)	n (%)			
NR3C1	CC	108 (54)	122 (61)	1.469	0.623–3.466	0.380
	GC	78 (39)	68 (34)	1.192	0.495–2.871	0.695
	GG	14 (7)	10 (5)	1.371	0.594–3.163	0.531
Allele						
	C	294 (73.5)	312 (78)			
	G	106 (26.5)	88 (22)	1.264	0.914–1.747	0.162

^&^Fisher’s exact test

In the patients who had undergone ASCT, the AA genotype of UCP-2 was also significantly more common (p = 0.001), while the GG genotype was significantly more common in the healthy control group (p = 0.016) **(**Additional file 1: Table S1).

In the analysis performed for pre-ASCT response assessment, no difference was found between the patients with at least a PR response and the other subgroup in terms of UCP-2 genotype distribution (p > 0.05 for all) (Additional file 1: Table S2).

In Additional file 1: Table S3, the results of multivariate analysis to show statistical significance are provided. It was found that having the GG genotype of the UCP-2 gene was a 2.48-fold risk factor (HR: 0.403, 95% CI: 0.163–0.993, p = 0.040).

No significant relationship was found between ISS stages and genotypes **(**Additional file 1: Table S4). There was no statistically significant factor found to affect PFS. OS was found to be significantly shorter in patients with the UCP-2 GG genotype (p = 0.034) (Additional file 1: Table S5).

Patients who underwent ASCT were divided into subgroups according to their ISS stages and survival, with the comparison illustrated in Additional file 1: Figure S1. Although the survival of the patients in the stage II and III groups at the 36^th^ month of follow-up was similar, it was observed that the difference became significant in the long-term follow-up.

Five-year analysis in terms of survival was repeated based on the different alleles of the UCP-2 gene as shown in the previous tables. It was determined that patients with the AA or AG allele among the UCP-2 genotypes had 5-year OS with a median survival of 101.6 months and those with the GG genotype had 5-year OS with a median survival of 82.2 months (Additional file 1: Figure S2).

### Discussion

This study can be considered important research in terms of the limited availability of data on the relationships between UCP-2 and NR3C1 and MM. It should be emphasized that this work contains new data in many regards, particularly on the relationship between UCP-2 and MM.

In order to explain the relationship between UCP-2 and MM, research on topics other than ROS-related anticancer effects should be pursued. In this context, important results were obtained in a study examining the relationship between UCP-2 and the tumor microenvironment (TME) [19]. The importance of T cells in patients’ anticancer responses is important here; many patients lack a T cell response, which significantly affects the TME. UCP-2 expression increases the T cell response and has a positive effect on OS via the TME [19]. UCP-2 also reprograms the immunity of the TME by altering the cytokine environment in a manner dependent on interferon regulatory factor 5 [19]. UCP-2 enhances the conventional type 1 dendritic cell-dependent and CD8 + T cell-dependent anti-tumor immune cycle [19]. Additionally, it was shown that the induction of UCP-2 makes melanoma patients susceptible to cell death protein-1 blockade therapy and elicits effective anti-tumor responses. In this study, the UCP-2 AA genotype was found to be significantly more common in the MM group while the GG genotype was more common in the healthy control group. It may thus be suggested that having the AA genotype is associated with the risk of developing MM. Similarly, the fact that the GG genotype was significantly more common among healthy controls suggests that this genotype is a protective factor against developing MM. The fact that OS was significantly shorter in patients with the UCP-2 GG genotype is an important finding and OS is defined as a risk factor for mortality. Not only treatment-related response but also factors effective in terms of tumor immunity and TME should be taken into consideration.

Cancer biogenetics involves a complex biological metabolism. UCP-2 and cancer-biology relationship also includes a similar complicated structure. G allele of the UCP2-866G/A polymorphism has lower UCP-2 mRNA/protein expression levels compared to the A allele, resulting in increased ROS generation [20]. Although this suggests that the UCP2 A allele has anti-cancer properties; literature data point to different results. In head and neck, skin, prostate, and pancreatic tumor samples, the protein levels of UCP2 were significantly higher in tumor tissues than that in the adjacent normal tissues; but the protein levels of UCP2 was lower in non-small cell lung tumor tissues [21]. When the survival analyzes were examined, the G allele was found to be associated with short survival, especially in colon tumors [22]. It seems possible to associate this condition with treatment. Patients with relatively low UCP2 expression are more sensitive to chemotherapeutic agents and have a better survival rate. A potential mechanism of UCP2-caused chemoresistance is a reduction in the generation of ROS.While this mechanism has been proven for gemcitabine, doxorubicin and cisplatin [22], further studies are needed for agents used in the treatment of MM.

Looking at the responses obtained before ASCT, the fact that there was no significant difference between the group with the lowest rate of PR and the other subgroup in terms of UCP-2 genotype distribution reveals that the UCP-2 GG genotype did not affect the treatment responses before ASCT but had a very important role in the follow-up period after ASCT.

Unlike UCP-2, NR3C1 has been the subject of many studies on hematological malignancies, specifically in cases of hematological tumors where steroid-based therapies are widely used. In a study on dexamethasone-based MM treatment and NR3C1 [23], the clinical effect of expression levels of NR3C1 was investigated with gene expression profiling (GEP) for 351 patients with initial GEP data and 130 patients with relapse. Low NR3C1 expression levels had a negative impact on PFS and OS in the thalidomide-free arm of that study. Post-relapse survival was adversely affected by low NR3C1 levels in the multivariate analysis in terms of both baseline and relapse parameters.

GR levels can be autologously regulated by its ligand and by transcriptional, post-transcriptional, and post-translational mechanisms. In a study in which GC resistance was examined [24], a gradual decrease in NR3C1 transcripts was seen during the development of resistance. Although important results were obtained regarding the regulation of GR expression, no effects on PFS or OS were detected in the same study. In the present study, neither a significant result between MM and the healthy control group nor any effect on PFS and OS could be detected.

In conclusion, the AA genotype of UCP-2 was found to be associated with the risk of developing MM. Conversely, the fact that the GG genotype was significantly more common in healthy controls suggests that this genotype is a protective factor against the development of MM. The fact that OS is significantly shorter with the UCP-2 GG genotype and its definition as a risk factor for mortality in MM have been put forth here for the first time in the literature. It may be among the high-risk cytogenetic factors and should be considered as a part of the approach for patients with high cytogenetic risk. New prospective studies will be necessary to confirm this.

### Limitations

While making an important contribution to the literature, this study also had limitations. UCP-2 and NR3C1 were isolated from all cells, not specifically from malignant plasma cells. The fact that the change in expression was not determined after treatment can be seen as another limitation. Although the GG genotype of UCP-2 did not play a role in pre-transplant response, it is noteworthy that the GG genotype did show differences in terms of OS. This finding needs to be confirmed by further studies, because the post-transplant treatment differences here were not sufficient for optimal statistical evaluation.

## Supplementary Information

**Additional file 1**: **Table S1**. Comparison of UCP-2 gene variants between MM patients who underwent ASCT and healthy control group. **Table S2**. Comparison of UCP-2 gene variants between response subgroups before ASCT. **Table S3**. Multivariate analysis of patients who underwent ASCT. **Table S4**.Comparison of frequencies of UCP-2 gene variants between ISS stages. **Table S5**. Comparison of PFS and OS with prognostic factors of patients who underwent ASCT. **Figure S1**. The 5-year overall survival (OS) of patients who underwent ASCT according to ISS stages. **Figure S2**. The 5-year overall survival (OS) of patients who underwent ASCT according to UCP-2 genotypes.

## Data Availability

The datasets analysed during the current study are available on [figshare], under https://doi.org/10.6084/m9.figshare.13229141.v1.

## Abbreviations

MM: Multiple myeloma
ASCT: Autologous stem cell transplantation
β2-MG: Beta-2 microglobulin
ISS: International staging system
LDH: Lactate dehydrogenase
FISH: Fluorescent in situ hybridization
Del: Deletion
UCP-2: Uncoupling protein-2
NR3C1: Nuclear receptor subfamily 3 group C member 1
ROS: Reactive oxygen species
GR: Glucocorticoid receptor
ECOG: Eastern Cooperative Oncology Group
CRP: C-reactive protein
OS: Overall survival
PFS: Progression-free survival
VCD: Bortezomib, cyclophosphamide, and dexamethasone
LD: Lenalidomide and dexamethasone
TME: Tumor microenvironment
GEP: Gene expression profiling
