# Supplementary material for: Effect of the uncoupling protein-2 (UCP-2) and nuclear receptor subfamily 3 group C member 1 (NR3C1) genes on treatment efficacy and survival in patients with multiple myeloma: a single-center study
Source: BMC Res Notes. 2021 Sep 4;14:346. doi: 10.1186/s13104-021-05758-7 (PMC8418283; doi:10.1186/s13104-021-05758-7)
Supplement: Supplementary file 1 — Additional file 1: Table S1. Comparison of UCP-2 gene variants between MM patients who underwent ASCT and healthy control group. Table S2. Comparison of UCP-2 gene variants between response subgroups before ASCT. Table S3. Multivariate analysis of patients who underwent ASCT. Table S4.Comparison of frequencies of UCP-2 gene variants between ISS stages. Table S5. Comparison of PFS and OS with prognostic factors of patients who underwent ASCT. Figure S1. The 5-year overall survival (OS) of patients who underwent ASCT according to ISS stages. Figure S2. The 5-year overall survival (OS) of patients who underwent ASCT according to UCP-2 genotypes. [file 13104_2021_5758_MOESM1_ESM.docx]

**Table S1. Comparison of UCP-2 gene variants between MM patients who underwent ASCT and healthy control group**

|  | **Genotype** | **MM**  **ASCT** | **Healthy Controls** | **OR**  **Exp(B)** | **95% CI** | **p^&^** |
| --- | --- | --- | --- | --- | --- | --- |
|  |  | n (%) | n (%) |  |  |  |
| **UCP-2** | AA | 71 (45.8) | 32 (16) | 0.201 | 0.101-0.401 | **0.001** |
|  | AG | 66 (42.6) | 125 (62.5) | 0.812 | 0.433-1.522 | 0.515 |
|  | GG | 18 (7.6) | 43 (21.5) | 0.480 | 0.264-0.871 | **0.016** |
| **Allele** |  |  |  |  |  |  |
|  | A | 208 (67.1) | 189 (47.25) |  |  |  |
|  | G | 102 (32.9) | 211 (52.75) | 0.439 | 0.323-0.597 | **0.001** |
| MM: Multiple myeloma; ASCT: autologous stem cell transplantation; OR: odds ratio (95% confidence interval (CI)); ^&^Fisher’s exact test, p < 0.05 | | | | | | |

**Table S2. Comparison of UCP-2 gene variants between response subgroups before ASCT**

|  | **Genotype** | **At least a PR** | **No response** | **OR**  **Exp(B)** | **95% CI** | **p^&^** |
| --- | --- | --- | --- | --- | --- | --- |
|  |  | n (%) | n (%) |  |  |  |
| **UCP-2** | AA | 71 (45.8) | 21 (46.7) | 1.645 | 0.419-3.761 | 0.446 |
|  | AG | 66 (42.6) | 20 (44.4) | 1.559 | 0.363-3.858 | 0.640 |
|  | GG | 18 (7.6) | 4 (9.9) | 1.641 | 0.304-3.646 | 0.689 |
| **Allele** |  |  |  |  |  |  |
|  | A | 208 (67.1) | 62 (68.9) |  |  |  |
|  | G | 102 (32.9) | 28 (31.1) | 1.787 | 0.344-2.919^&^ | 0.531^&^ |
| ASCT: Autologous stem cell transplantation; OR: odds ratio (95% confidence interval (CI)); ^&^Fisher’s exact test, p < 0.05 | | | | | | |

**Table S3. Multivariate analysis of patients who underwent ASCT**

|  |  |  | **OS** |  |
| --- | --- | --- | --- | --- |
|  |  | **Exp(B)**  **Relative Risk** | **95% CI** | **p** |
| **Age** | <65 / ≥65 | 0.264 | 0.088-0.791 | **0.017** |
| **ISS** | I/II |  |  |  |
|  | III | 1.668 | 0.170-0.971 | **0.023** |
| **ECOG** | ≤ 1 / >1 | 0.432 | 9.094-19.84 | 0.281 |
| **LDH (IU/L)** | <480 / ≥480 | 0.630 | 0.162-2.446 | 0.505 |
| **CRP (mg/L)** | <5 / ≥ 5 | 0.556 | 0.196-1.579 | 0.270 |
| **UCP-2** | AA/AG |  |  |  |
|  | GG | 0.403 | 0.163-0.993 | **0.040** |

ASCT: Autologous stem cell transplantation; OS: overall survival; ECOG: Eastern Cooperative Oncology Group performance status; CRP: C-reactive protein; LDH: lactate dehydrogenase; ISS: International Staging System

**Table S4. Comparison of frequencies of UCP-2 gene variants between ISS stages**

|  | ***UCP-2***  **GG** | ***UCP-2***  **AA/AG** | **OR***  **Exp(B)** | **95% CI** | **p^&^** |
| --- | --- | --- | --- | --- | --- |
| **ISS** | n (%) | n (%) |  |  |  |
| I | 4 (25) | 37 (33) | 1.458 | 0.392-5.427 | 0.574 |
| II | 5 (31.3) | 30 (26.8) | 0.892 | 0.250-3.183 | 0.860 |
| III | 7 (43.7) | 45 (40.2) | 0.864 | 0.300-2.486 | 0.792 |
| *Odds ratio (OR) (95% confidence interval (CI)) was adjusted by age and sex, ^&^Fisher’s exact test | | | | | |

**Table S5. Comparison of PFS and OS with prognostic factors of patients who underwent ASCT**

|  |  |  | **PFS** | **Log Rank**  **p-value** | **OS** | **Log Rank**  **p-value** |
| --- | --- | --- | --- | --- | --- | --- |
|  |  | **n** | 54.3 |  | 77 |  |
| **Gender, n** | Female / Male | 74 / 81 | 89.6 / 43.8 | 0.057 | 96 / 54 | 0.147 |
| **Age, n** | <65 / ≥65 | 131 / 24 | 69.3 / 40.4 | 0.476 | 97.3 / 46 | **0.001** |
| **Stage (Durie-Salmon), n** | II / III | 38 / 89 | 54.3 / 47.1 | 0.580 | 99.1 / 87.7 | 0.313 |
|  | A / B | 96 / 31 | 72.2 / 43.8 | 0.268 | 74 / 33 | 0.332 |
| **ISS, n** | I | 51 | 73.1 |  | 88 |  |
|  | II | 43 | 47.1 |  | 66 |  |
|  | III | 61 | 44.4 | 0.616 | 54 | **0.001** |
| **Ig subtype, n** | k / l | 99 / 56 | 54.3 / 44.4 | 0.663 | 94 / 87.1 | 0.473 |
|  | Light chain | 28 | 73.1 | 0.704 | 88 | 0.770 |
| **ECOG, n** | ≤ 1 / >1 | 129 / 26 | 54.3 / 28.5 | 0.852 | 79 / 33 | **0.004** |
| **Thrombocytes (×10^3^/mm^3^), n** | <150 / ≥150 | 26 / 129 | 27.4 / 38.1 | 0.563 | 53.1 / 99.1 | 0.179 |
| **LDH (IU/L), n** | <480 / ≥480 | 135 / 20 | 54.3 / 17 | 0.067 | 67.7 / 17 | **0.001** |
| **CRP (mg/L), n** | <5 / ≥ 5 | 67 / 88 | 54.3 / 52.7 | 0.946 | 98.7 / 87.1 | **0.008** |
| **NR3C1, n** | CC | 86 | 52.7 |  | 66.1 |  |
|  | GC | 57 | 54.3 |  | 56.6 |  |
|  | GG | 12 | 24.4 | 0.882 | 46.9 | 0.582 |
| **UCP-2, n** | AA/AG | 137 | 69.3 |  | 101.6 |  |
|  | GG | 18 | 39.2 | 0.550 | 82.2 | **0.034** |
| CD: Bortezomib, cyclophosphamide, dexamethasone; LD: lenalidomide, dexamethasone; ASCT: autologous stem cell transplantation; ECOG: Eastern Cooperative Oncology Group performance status; CRP: C-reactive protein; LDH: lactate dehydrogenase; IPI: International Prognostic Index; PFS: progression-free survival, OS: overall survival | | | | | | |


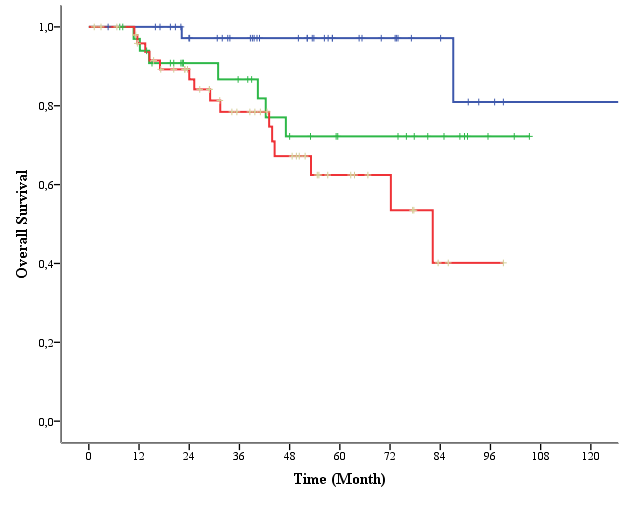


**ISS-II 5-year OS: 72%**

**ISS-III 5-year OS: 62%**

**ISS-I 5-year OS: 81%**

**Figure S1. The 5-year overall survival (OS) of patients who underwent ASCT according to ISS stages**

**
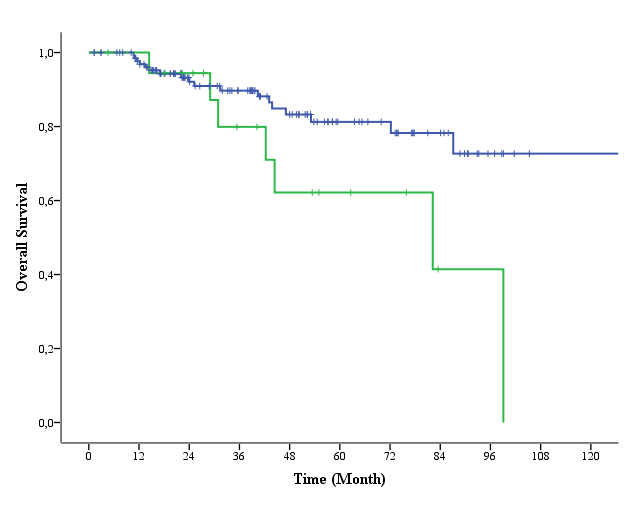
**

**UCP-2 AA/AG 5-year OS (median: 101.6)**

**UCP-2 GG 5-year (median: 82.2 months)**

**Figure S2. The 5-year overall survival (OS) of patients who underwent ASCT according to UCP-2 genotypes**
